# Primary Biliary Cirrhosis with a normal Alkaline Phosphatase: a case report

**DOI:** 10.1186/1757-1626-1-33

**Published:** 2008-07-14

**Authors:** Joshua M Brostoff, Sukaina Rashid, Daniel McCrea

**Affiliations:** 1 34 Fitzjohns Avenue, London , NW3 5NB, UK; 2 Central Middlesex Hospital, London, NW10 7NS, UK

## Abstract

A 78 year-old lady presented with abdominal swelling and fatigue. She was anaemic with mild hypoalbuminaemia, and had a normal alkaline phosphatase. Computed tomography showed hepatosplenomegaly and mild ascites. Anti mitochondrial antibodies were strongly positive, as were anti nuclear antibodies, and the gamma glutamyl-transferase was shown to be elevated. A diagnosis of primary biliary cirrhosis was made. A brief discussion of treatment of primary biliary cirrhosis follows. The case is notable for the fact that primary biliary cirrhosis can manifest clinically without an elevation in alkaline phosphatase – normally the hallmark of the disease.

## Case presentation

A 78 year-old lady of West Indian origin presented with a 4 week history of diarrhoea and progressive abdominal swelling, as well as shortness of breath and poor mobility over the same period. She denied any other symptoms, foreign travel, infectious contacts, or family history of note. Her past medical history included hypertension, glaucoma, and well-controlled type two diabetes mellitus. Medications were bendrofluazide, gliclazide and metformin, and she had no allergies. She was unmarried and had no children. The patient was a non-smoker, and drank one small glass of whisky per day.

Physical examination revealed a grossly distended, non-tender abdomen with shifting dullness. There was no palpable organomegaly, and bowel sounds were normal. A digital rectal exam showed watery mucus but no masses. There was pedal oedema bilaterally, and fine inspiratory crepitations audible at both lung bases.

Initial laboratory investigations revealed a haemoglobin (Hb) of 9.0 g/dl (NR 11.5–15.5) with a mean corpuscular volume (MCV) of 86 fl (NR 78–100) but otherwise normal haematological indices. The biochemical and clotting profiles were normal apart from an albumin of 28 g/L (NR 35–50), and thyroid function tests were normal. CT scanning revealed hepatosplenomegaly and moderate ascites but no pleural effusions, and echocardiography showed moderate diastolic dysfunction with structurally normal valves.

Further blood results returned showing a gamma-glutamyl transferase (GGT) of 140 IU/L (NR 9–36), and a polyclonal increase in gammaglobulins with normal IgM fraction. Autoimmune profile showed strongly positive anti-mitochondrial antibodies, and anti-nuclear antibodies staining in a centromere pattern. A diagnosis of PBC was made and the patient was started on ursodeoxycholic acid. In view of the patient's age and frailty a liver biopsy was not undertaken.

## Discussion

Primary biliary cirrhosis (PBC) is a presumed autoimmune disease of the liver, which is most commonly diagnosed in women between the ages of 30 and 65. In the modern era most cases are diagnosed when asymptomatic, usually presenting with abnormal LFTs. Symptoms can include fatigue, pruritus, rheumatic symptoms, skin hyperpigmentation, and right upper quadrant pain [1].

The antimitochondrial antibody is the serological hallmark of disease and is present in the serum of nearly all affected patients [2]; likewise the serum alkaline phosphatase (ALP) level is almost always raised, often to striking levels [3].

Treatment is aimed at alleviating the symptoms and complications of the disease, and attempting to suppress the continuing destruction of small bile ducts. Ursodeoxy-cholic acid (UDCA) may work by suppressin release of endogenous bile acids which are toxic to hepatocytes, and it also inhibits eosinophil activation, which may have a pathogenic role in PBC. UDCA at approximately 13 to 15 mg/kg per day has been studied extensively in individual trials and has been the subject of at least 3 meta-analyses [4]. Results of these are conflicting, but considering the results of the individual trials, the few effective available alternative treatments for patients with PBC, and the excellent tolerability of UDCA, it is still widely considered first line therapy.

This case is unusual for several reasons. The patient was diagnosed at an older age than is usual with PBC. She had unusual presenting symptoms, with ascites as her chief complaint. Most important, this case highlights that PBC can exist with an entirely normal ALP, and here the diagnosis hinged on a high index of suspicion and an elevated GGT.

At our hospital GGT is not included in routine liver function tests, and needs to be specifically requested. It was only after the elevated GGT was noted despite the normal ALP that we began to strongly suspect cholestasis and therefore PBC. Hence we suggest that GGT is always included in routine liver function tests.

## Abbreviations

ALP: Alkaline Phosphatase. CT: Computed Tomography. GGT: Gamma Glutamyl-Transferase. Hb: Haemoglobin. LFT: Liver Function Tests. MCV: Mean Corpuscular Volume. NR: Normal Range. PBC: Primary Biliary Cirrhosis. UDCA: Urso Deoxycholic Acid.

## Competing interests

The authors declare that they have no competing interests.

## Authors' contributions

Case report written by SR and JMB. Discussion written by JMB and DM.

## Consent

The patient has died since the original presentation, and leaves behind no next-of-kin.

## References

[B1] Prince MI, Chetwynd A, Craig WL, Metcalf JV, James OF (2004). Asymptomatic primary biliary cirrhosis: clinical features, prognosis, and symptom progression in a large population based cohort. Gut.

[B2] Water J Van de, Cooper A, Surh CD, Coppel R, Danner D, Ansari A, Dickson R, Gershwin ME (1989). Detection of autoantibodies to recombinant mitochondrial proteins in patients with primary biliary cirrhosis. N Engl J Med.

[B3] Christensen E, Crowe J, Doniach D, Popper H, Ranek L, Rodes J, Tygstrup N, Williams R (1980). Clinical pattern and course of disease in primary biliary cirrhosis based on an analysis of 236 patients. Gastroenterology.

[B4] Silveira MG, Lindor KD (2008). Treatment of primary biliary cirrhosis: therapy with choleretic and immunosuppressive agents. Clin Liver Dis.

